# Glycan Node Analysis Detects Varying Glycosaminoglycan Levels in Melanoma-Derived Extracellular Vesicles

**DOI:** 10.3390/ijms24108506

**Published:** 2023-05-09

**Authors:** Jenifer Pendiuk Goncalves, Sierra A. Walker, Jesús S. Aguilar Díaz de león, Yubo Yang, Irina Davidovich, Sara Busatto, Jann Sarkaria, Yeshayahu Talmon, Chad R. Borges, Joy Wolfram

**Affiliations:** 1 Australian Institute for Bioengineering and Nanotechnology, The University of Queensland, Brisbane, QLD 4072, Australia; j.pendiukgoncalves@uq.edu.au; 2 Department of Biochemistry and Molecular Biology, Department of Physiology and Biomedical Engineering, Department of Transplantation, Mayo Clinic, Jacksonville, FL 32224, USA; 3 School of Molecular Sciences and Virginia G. Piper Center for Personalized Diagnostics, The Biodesign Institute at Arizona State University, Tempe, AZ 85287, USA; 4 Department of Chemical Engineering and the Russell Berrie Nanotechnology Institute (RBNI), Technion-Israel Institute of Technology, Haifa 3200003, Israel; 5 Vascular Biology Program, Boston Children’s Hospital, Boston, MA 02115, USA; 6 Department of Surgery, Harvard Medical School, Boston, MA 02115, USA; 7 Department of Radiation Oncology, Mayo Clinic, Rochester, MN 55902, USA; 8 School of Chemical Engineering, The University of Queensland, Brisbane, QLD 4072, Australia

**Keywords:** extracellular vesicle, glycosylation, hyaluronic acid, mass spectrometry, melanoma

## Abstract

Extracellular vesicles (EVs) play important roles in (patho)physiological processes by mediating cell communication. Although EVs contain glycans and glycosaminoglycans (GAGs), these biomolecules have been overlooked due to technical challenges in comprehensive glycome analysis coupled with EV isolation. Conventional mass spectrometry (MS)-based methods are restricted to the assessment of N-linked glycans. Therefore, methods to comprehensively analyze all glyco-polymer classes on EVs are urgently needed. In this study, tangential flow filtration-based EV isolation was coupled with glycan node analysis (GNA) as an innovative and robust approach to characterize most major glyco-polymer features of EVs. GNA is a molecularly bottom-up gas chromatography-MS technique that provides unique information that is unobtainable with conventional methods. The results indicate that GNA can identify EV-associated glyco-polymers that would remain undetected with conventional MS methods. Specifically, predictions based on GNA identified a GAG (hyaluronan) with varying abundance on EVs from two different melanoma cell lines. Enzyme-linked immunosorbent assays and enzymatic stripping protocols confirmed the differential abundance of EV-associated hyaluronan. These results lay the framework to explore GNA as a tool to assess major glycan classes on EVs, unveiling the EV glycocode and its biological functions.

## 1. Introduction

Extracellular vesicles (EVs) are membrane-surrounded nanoparticles released by all cells [1]. EVs are essential for local and systemic intercellular communication and inherit molecular cargo from parental cells. EVs are also involved in pathological settings, such as inflammation [2], infections [3], neurological diseases [4], and cancer [5]. The comprehensive molecular characterization of EVs is important for improving mechanistic understanding and promoting diagnostic [6,7], therapeutic [8,9,10,11], and drug delivery applications [12,13,14,15].

Glyco-polymers, including glycans, glycoproteins, glycolipids, and glycosaminoglycans (GAGs), are essential for cell functions and communication. In addition to intracellular glyco-polymers, cells are covered by an outer glycocalyx that mediates interactions between cells and the microenvironment [16]. Therefore, glyco-polymer analysis is critical for understanding biological processes and developing therapeutic and diagnostic strategies. For example, altered glycosylation patterns have been linked to cancer progression, leading to the clinical approval of several glycoproteins or carbohydrate antigens as cancer biomarkers [17]. Additionally, natural and engineered glycans are key components of several commercial therapeutic products, such as antibiotics, anticoagulants, vaccines, antibodies, and nutritional supplements [18].

Although glyco-polymers are major components of EVs, these biomolecules are often overlooked due to technical challenges in EV isolation coupled with comprehensive and high-throughput glycomics [19]. In recent years, studies focused on the characterization and function of EV glycans have become more common [20,21,22,23,24]. However, most of these studies focus exclusively on N-glycans (glycan attachment to an asparagine residue of a protein) [25], overlooking other glyco-polymer classes.

To enable a more comprehensive assessment of glyco-polymers, a bottom-up gas chromatography-mass spectrometry (MS)-based approach, termed glycan node analysis (GNA), was developed. This method involves glyco-polymer methylation, degradation, and acetylation, enabling the analysis of individual monosaccharides, specific linkages, and branch points (glycan nodes) as single analytical signals that correspond to their existence as spread across a number of highly heterogenous intact polymer molecules [26,27,28]. This approach has previously been used to analyze biofluids from physiological and pathological conditions, such as cancer, for the identification of biomarker candidates [29,30,31]. Recently, we applied GNA to assess glycan features of plasma-derived EVs [32] and endothelial-derived EVs [33], demonstrating that inflammatory stimuli increase terminal galactose on EVs [33]. However, the utility of GNA in accurately predicting whole EV glyco-polymers based on branch points remains unknown.

The aim of this study was to demonstrate the innovative application of GNA for the comprehensive detection of changes in glycan features in EV samples with the subsequent validation of changes in whole glyco-polymers using affinity-based molecular assays. Two melanoma cell lines (non-metastatic and metastatic) were used in the study, as the simultaneous characterization of multiple classes of glyco-polymers on melanoma-derived EVs has not previously been reported. The suitability of these cell lines for the study is also inferred by likely differences in EV glyco-polymer characteristics, as EVs are known to facilitate metastasis [5] and glycan alterations are common during melanoma progression [34]. Previous studies also indicated that EVs from these cell lines display distinct functional characteristics, such as labeling using lipophilic probes and interactions with and uptake by monocytes, triggering distinct cytokine production [35,36], suggesting differences in the molecular composition. This is the first study to demonstrate that glycan node data can be used to predict the differential abundance of a GAG.

## 2. Results

**Figure 1:**
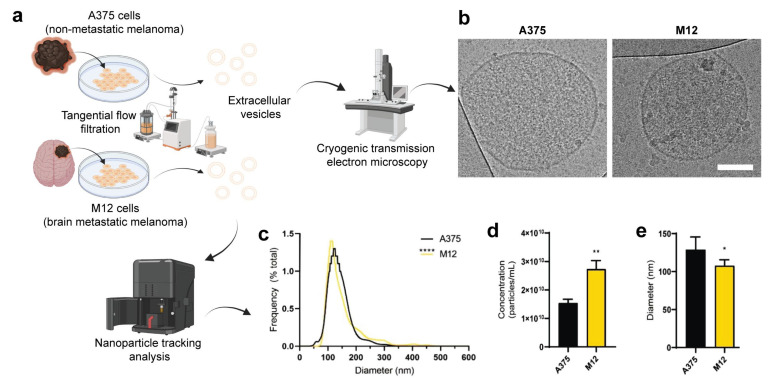
Physicochemical characterization of extracellular vesicles (EVs) from human A375 melanoma cells and human M12 melanoma brain metastases cells. (**a**) Schematic representation of the origin and purification of A375 and M12 cell lines. (**b**) Cryogenic transmission electron microscopy images. Scale bar, 100 nm. (**c**) Size distribution profiles of EVs. (**d**) Concentration of EVs. (**e**) Average modal diameter of each sample. Data are presented as the mean (unless otherwise noted) + SD of five replicates. Statistics are based on a non-parametric Mann-Whitney test. *, *p* < 0.05; **, *p* < 0.01; ****, *p* < 0.0001.

**Figure 2:**
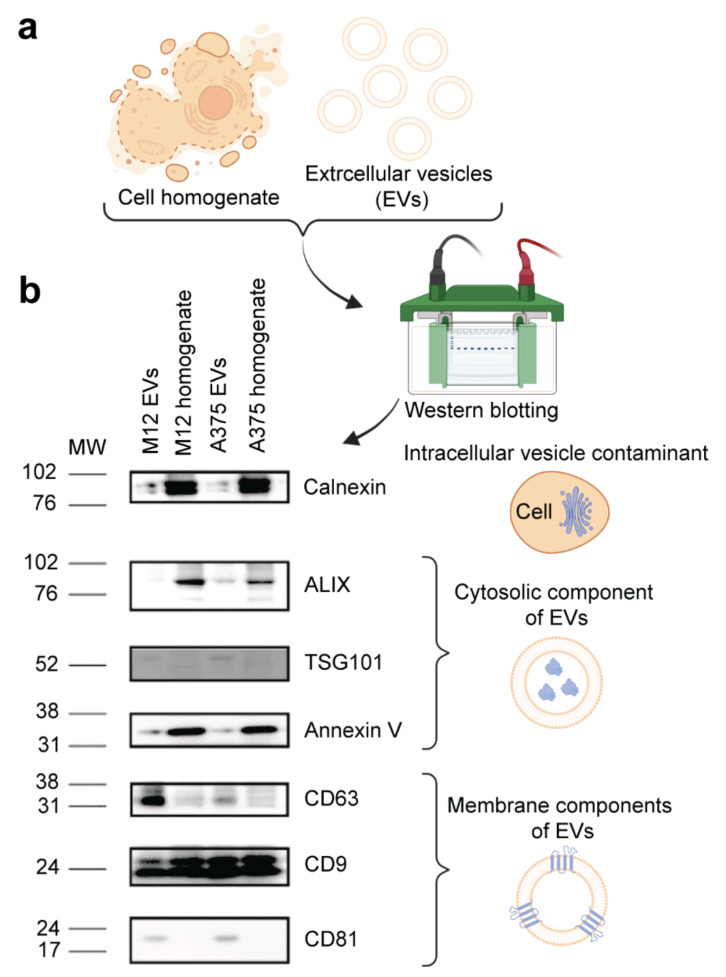
Molecular characterization of EVs from human A375 melanoma cells and human M12 melanoma brain metastases cells. (**a**) Schematic representation of samples. (**b**) Western blot images of intracellular vesicle contaminant marker calnexin and EV cytosolic (ALIX, TSG101, and annexin V) and membrane (CD63, CD9, and CD81) markers.

EVs from two human melanoma cell lines (non-metastatic A375 and brain metastatic M12) (Figure 1a) were isolated using TFF, which is a method for obtaining EVs with a high yield and purity from cell culture [37]. Several techniques were used to authenticate and characterize EVs. For example, cryogenic transmission electron microscopy (cryo-TEM) was used for its unique ability to assess the morphological features of single EVs [38], confirming that the melanoma cell-derived EVs had a lipid bilayer structure (Figure 1b). The size distribution profiles of EVs assessed via nanoparticle tracking analysis were within the expected range (50–300 nm) (Figure 1c,e). Additionally, the M12 cell line produced more EVs in comparison with the A375 cell line (Figure 1d), a phenomenon that has previously been demonstrated in other studies and is correlated to worse cancer patient survival [39,40]. Finally, Western blotting revealed that two EV membrane markers (CD63 and CD81) were enriched in EV samples compared to levels in corresponding cell homogenates, while the intracellular vesicle contaminant marker, calnexin, was more abundant in cell homogenates (Figure 2a,b). Additional protein markers, including CD9, annexin V, ALIX, and TSG101 were present in the EV samples to varying extents but were not enriched compared to levels in the corresponding cell homogenates (Figure 2b). These data demonstrate that EVs from both cell lines were successfully authenticated in accordance with guidelines from the International Society of Extracellular Vesicles [41].

**Figure 3:**
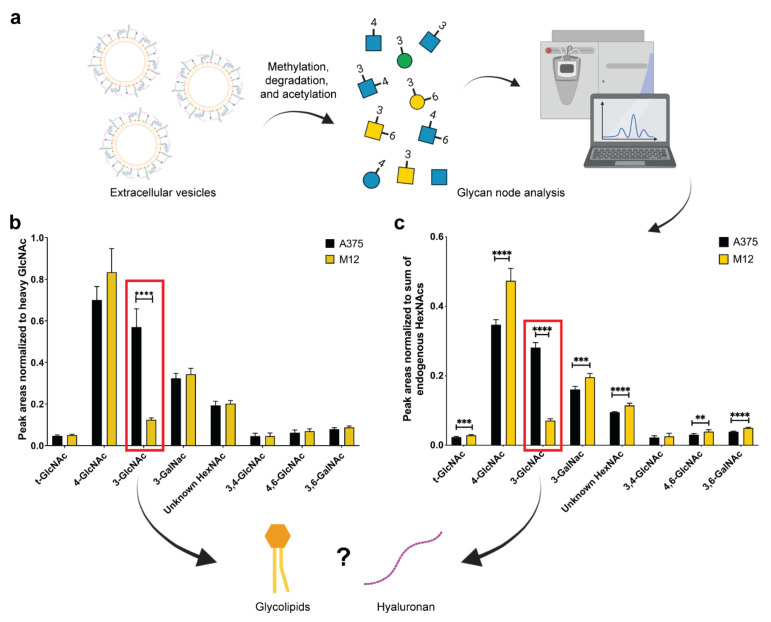
Glycan node analysis of EVs from human A375 melanoma cells and human M12 melanoma brain metastases cells. (**a**) Schematic representation of EV glycan node analysis. (**b**) Peak areas normalized to heavy N-acetylglucosamine. (**c**) Peak areas normalized to the sum of endogenous N-acetylhexosamines (HexNAcs). Red squares highlight significant changes in 3-linked N-acetylglucosamine (3-GlcNAc). All data are presented as the mean ± standard deviation (SD) of six replicates. For each glycan node, differences between EV subtypes were searched for with a Student’s *t*-test, using the two-stage linear step-up procedure of Benjamini, Krieger, and Yekutieli, with Q = 0.1% to correct for false discoveries. **, *p* < 0.01; ***, *p* < 0.001; ****, *p* < 0.0001.

To assess differences in the glycan nodes of A375 and M12 melanoma cell-derived EVs, GNA was performed to analyze hexoses and N-acetylhexosamines (HexNAcs) (Figure 3a). As shown in Figure 3b,c, statistically significant differences in several HexNAcs were detected. The most substantial difference between the EV samples, regardless of the normalization strategy (heavy N-acetylglucosamine versus the sum of endogenous HexNAcs), was in 3-linked N-acetylglucosamine (3-GlcNAc). In particular, EVs from the A375 cell line displayed a 3.6-fold (Figure 3b) and 2.9-fold (Figure 3c) enrichment of 3-GlcNAc compared to EVs from the M12 cell line. A previous study found that differences in 3-GlcNAc can be attributed to glycolipids and/or hyaluronan [26].

**Figure 4:**
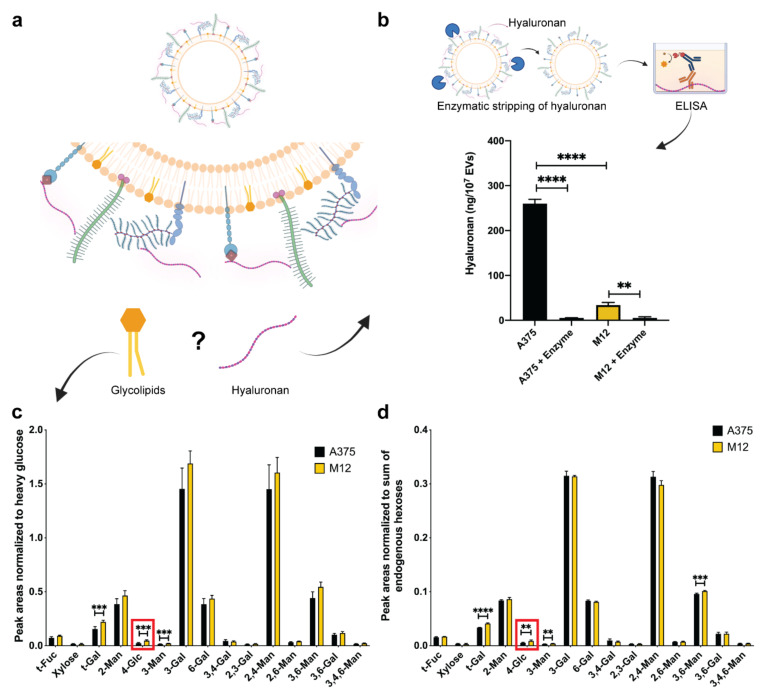
Validation of the origin of 3-GlcNAc changes in EVs from human A375 melanoma cells and human M12 melanoma brain metastases cells. (**a**) Schematic representation of extracellular vesicle-associated hyaluronan and glycolipids as potential sources of 3-GlcNAc. (**b**) Enzyme-linked immunosorbent assay (ELISA) measurements of hyaluronan levels with and without enzymatic digestion with hyaluronidase. Data represent the mean + SD of triplicates. Statistical analysis was performed by ANOVA with post-hoc pairwise comparisons calculated with a Sidak’s test. **, *p* < 0.01; ****, *p* < 0.0001. (**c**) Glycan node analysis peak areas normalized to heavy glucose, and (**d**) to endogenous hexoses. Red squares highlight significant changes in 4-linked glucose (4-Glc). Data are presented as the mean ± standard deviation (SD) of six replicates. For each glycan node, differences between EV subtypes were searched for with a Student’s *t*-test, using the two-stage linear step-up procedure of Benjamini, Krieger, and Yekutieli, with Q = 0.1% to correct for false discoveries. **, *p* < 0.01; ***, *p* < 0.001; ****, *p* < 0.0001.

To assess whether differing levels of 3-GlcNAc in EVs from the two melanoma cell lines were due to hyaluronan and/or glycolipids, further analysis was performed. Specifically, an enzyme-linked immunosorbent assay (ELISA) was used to determine whether differences in 3-GlcNAc levels were due to hyaluronan. The results demonstrated that the A375 melanoma EVs displayed a 6.6-fold increase in hyaluronan compared to the M12 melanoma EVs (Figure 4b). Enzymatic stripping of EVs incubated with hyaluronidase depleted hyaluronan in both EV samples (Figure 4b), further confirming the identity of this GAG quantified in both samples. Additionally, GNA results of endogenous hexoses demonstrated that the difference in 4-linked glucose (4-Glc) was less evident between the two samples compared to what was observed for 3-GlcNAc. Specifically, a 0.48-fold (Figure 4c) and 0.4-fold (Figure 4d) difference was observed for 4-Glc depending on whether heavy glucose or endogenous hexoses, respectively, were used as a normalization strategy. Therefore, the substantial fold-change in 3-GlcNAc accompanied by a much less evident fold-change in 4-Glc, together with the ELISA results, confirms a difference in the abundance of hyaluronan in the two EV samples.

## 3. Discussion

Hyaluronan is synthesized as various molecular weights, ranging from 100 kDa up to 4000 kDa, and is broken down to smaller fragments post-synthesis by hyaluronidases in the extracellular space [42,43]. Although cancer is usually associated with low-molecular-weight hyaluronan [44], it is worth noting that non-EV bound high-molecular-weight hyaluronan may be present in the samples as a co-isolated contaminant.

The skin is highly enriched in hyaluronan, which contributes to a flexible and hydrophilic extracellular matrix that facilitates cell migration, proliferation, and cell communication during physiological processes, such as wound healing [45]. Hyaluronan expression in melanoma is complex and tends to be higher during the initial stages of disease progression, with advanced stages displaying enhanced degradation of this GAG [46,47]. While the production of hyaluronan by human melanoma cells correlates with enhanced migration in vitro, the formation of large primary tumors, and high metastatic potential [48,49], reduced expression is indicative of progressive disease and poorer prognosis [50]. The results from this study demonstrate a significant reduction in hyaluronan in EVs derived from melanoma brain metastases cells, which correlates with the above-mentioned findings and the adaptation of metastatic cells to a different microenvironment, that is the brain. The aim of this study was to demonstrate the innovative application of GNA for the comprehensive detection of changes in glycan features in EV samples with subsequent validation of changes in whole glyco-polymers using affinity-based molecular assays. To gain an understanding of the role of EV hyaluronan in melanoma metastasis, a broader range of cell lines and melanoma patient samples is required. Therefore, this study is limited regarding biological insight into the role of EV-associated hyaluronan in melanoma, which should be assessed in future studies.

In this study, GNA proved to be critical for detecting a dramatic change in hyaluronan in EVs from two melanoma cell lines. Conventional glycomics approaches generally focus on the N-linked glycosylation of proteins [51], while GAGs are often overlooked, indicating a clear gap in the comprehensive analysis of EV glyco-polymer classes. Alterations in individual glycan nodes correlate with differential net glycosyltransferase activity [26]. In this study, a significant difference in 3-GlcNAc between EVs from two melanoma cell lines indicates varying hyaluronan synthase and/or hyaluronidase activity. The ELISA results confirmed the differential abundance of hyaluronan between the two samples (Figure 4b). Therefore, for the first time, a GNA-generated prediction of a whole GAG was validated with an molecular assay, demonstrating the potential of GNA for the high-throughput screening of all major glyco-polymer classes.

Taken together, GNA holds promise as an initial screening platform for the identification of changes in all major glycan features with the potential for subsequent validation of changes in whole glyco-polymers using molecular assays, such as ELISAs or lectin arrays. Potential applications of GNA as a high-throughput screening approach for glyco-polymers in EV samples include identifying disease-specific glycan patterns that can be validated for use in the diagnosis, prognosis, and monitoring of disease progression. GNA also provides a comprehensive initial platform for the identification of glycan features and subsequent validation of whole glycans that may have causative roles in EV-mediated pathological processes, leading to the potential identification of novel therapeutic targets. Previous studies have shown that changes in glycosylation patterns in cancer are promising targets for therapeutics and biomarkers [17]. Studies have reported increased levels of cancer cell-derived EVs in the blood of cancer patients compared to those in healthy individuals, indicating that they may be more abundant and accessible than cancer cells in body fluids [52]. This study highlights a platform with expanded capability in terms of assessing the glycan features of these EVs. Potential future applications include the design of customized treatment and vaccination plans based on personalized EV glycan signatures [53]. Studies have also shown that modifications in EV glycans alter biodistribution patterns [17,54], suggesting an additional application of GNA as an initial screening platform for the subsequent identification and validation of EV-associated glycans with beneficial cell- or tissue-specific targeting properties. The identification of a cell, tissue, or organotropic EV glycocode will provide opportunities for engineering EVs with site-specific properties for targeted drug delivery to improve the efficacy and safety of EV-based therapies.

## 4. Conclusions

In conclusion, this study shows, for the first time, that tangential flow filtration combined with GNA is a valuable approach for detecting changes in glyco-polymer features of EVs with the potential to subsequently validate changes in whole glyco-polymers, as demonstrated with hyaluronan.

## 5. Materials and Methods

### 5.1. Cell Culture

Human A375 melanoma cells (CRL-1619; ATCC) and human M12 melanoma brain metastases cells (Mayo Clinic Institutional Review Board approval: IRB#07-007623) were cultured and maintained in high glucose Dulbecco’s Modified Eagle’s Medium (DMEM) (Life Technologies, Carlsbad, CA, USA) supplemented with 10% fetal bovine serum (FBS) (Sigma, St. Louis, MO, USA), 1% penicillin/streptomycin (Gemini Bioproducts, West Sacramento, CA, USA), and 1% glutamine (Life Technologies) at 37 °C in 5% CO_2_.

For EV production, cells were seeded in 150 mm dishes with DMEM supplemented with 10% EV-depleted FBS (Exosome-depleted FBS; System Biosciences, Palo Alto, CA, USA) and cultured for 48 h until 90% confluency was obtained with at least 95% viability (Trypan blue).

### 5.2. EV Isolation via Tangential Flow Filtration (TFF)

The conditioned cell culture media (typically 800 mL) were centrifuged at 800× *g* for 30 min (Sorvall ST 16R centrifuge, Thermo Scientific, Grand Island, NY, USA) to remove cellular debris. A KrosFlo Research 2i Tangential Flow Filtration System (Spectrum Labs, Los Angeles, CA, USA) was used to concentrate and purify EVs, as previously described [55,56]. Briefly, supernatants from cell culture media were filtered through sterile and rehydrated hollow fiber polyethersulfone membranes (0.65 μm pores), and the permeate was further filtered through sterile and rehydrated hollow fiber polysulfone membranes (500 kDa molecular weight cutoff). The final retentate was diafiltrated six times with a clinical-grade cryoprotective buffer (5% sucrose, 50 mM Tris, and 2 mM MgCl—Lonza, #08-735B, Bend, OR, USA) [24] and concentrated to a final volume of 6–9 mL for functional studies. For GNA, diafiltration was performed six times in high performance liquid chromatography (HPLC)-grade water (Thermo Fisher Scientific, Waltham, MA, USA). Aliquots of 500 μL were prepared in low protein binding microtubes and stored at −80 °C until further analysis.

### 5.3. Nanoparticle Tracking Analysis (NTA)

The EV concentration and size distribution were assessed by performing NTA. EVs at 2–3 × 10^10^ EVs/mL were diluted (1:100) in phosphate buffer saline (PBS; pH 7.4; GE Healthcare, Chicago, IL, USA), and analysis was performed on a NanoSight NS300 (Software v3.3; Malvern Panalytical, Malvern, UK). Samples were measured under a continuous syringe pump flow rate of 40 μL/min with the camera level set to 12 or 13 and detection threshold to three.

### 5.4. Cryo-TEM

Approximately 3 μL of EVs (10^10^/mL) was placed on perforated carbon film-coated 200 mesh TEM grids in a controlled-environment vitrification system (CEVS) [57]. A filter paper mounted on a metal strip was used to blot away excess solution and obtain a thin liquid film, less than 300 nm thick. The TEM grids were plunged into freezing ethane (−183 °C) and then imaged and recorded at low electron exposure using an FEI (now Thermo Fisher Scientific, Waltham, MA, USA) Talos 200C high-resolution TEM and a Falcon III direct-imaging camera at −180 °C. A Volta phase-plate was used to enhance image contrast, and images were acquired at a 200 kV acceleration voltage [35,58].

### 5.5. Western Blot

The bicinchoninic acid (BCA) protein assay kit (Thermo Scientific, Waltham, MA, USA) was used to determine the samples’ protein concentrations. After normalization, sodium dodecyl-sulfate (6×) was added to a final concentration of 1×, and samples were boiled for five minutes at 90 °C. Proteins (16 µg) were loaded in each well of a 12% polyacrylamide gel in MOPS buffer, electrophoretically separated for 1.5–2 h at 120 V, and then transferred to nitrocellulose membranes at 200 mA for 1.5 h. Membranes were blocked at 4 °C overnight using 5% milk (*w*/*v*) in 1× Tris-buffered saline (TBS) with 0.1% Tween (TBST). Membranes were cut horizontally, and each strip was incubated with the following primary antibodies (1:500 in 1% milk in TBST *w*/*v*) at room temperature for two hours: cluster of differentiation (CD)9 (Invitrogen by Thermo Fisher Scientific, #10626D, Waltham, MA, USA), CD63 (Abcam, # ab134045, Waltham, MA, USA), CD81 (Santa Cruz, #sc- 166029, Dallas, TX, USA), ALG-2-interacting protein X (ALIX, Cell Signaling, #2171S, Danvers, MA, USA), calnexin (Abcam, # ab22595,), Annexin V (Abcam, #ab14196), and tumor susceptibility gene (TSG)101 (Abcam, #ab83). Membranes were then washed and incubated with the following secondary antibodies (1:3000 in 1% milk in TBST (*w*/*v*)) for one hour at room temperature: anti-rabbit and anti-mouse (Cell Signaling, #7074S and #7076S, respectively, Danvers, MA, USA). Membranes were washed again and developed using either the Pierce™ ECL Substrate kit or the SuperSignal™ West Femto kit (Thermo Scientific, #32109 and #34094, respectively, Waltham, MA, USA) depending on the signal intensity, which was captured using the myECL Imager (Thermo Scientific, Waltham, MA, USA). ImageJ (v1.52a, U. S. National Institutes of Health, Bethesda, MD, USA) was used to quantify the relative protein expression.

### 5.6. GNA

The GNA was adapted from previous publications [26,27,32]. Speed-vac-concentrated EV samples (10 μL; 10 mg/mL protein concentration) were fortified with 1 μL of 10 mM heavy, stable-isotope-labeled D-glucose (U−13C6, 99%; 1,2,3,4,5,6,6-D7, 97–98%; Cambridge Isotope Laboratories) and N-Acetyl-D-[UL-13C6] glucosamine (Omicron Biochemicals, Inc., South Bend, IN, USA) in water. Samples were then mixed with 270 μL of dimethylsulfoxide (DMSO, Sigma-Aldrich, St. Louis, MO, USA, 540 μL) and 105 μL of iodomethane (99%, Sigma-Aldrich, #I8507, St. Louis, MO, USA, 105 μL) by vortexing. Liquid samples were then mixed with ~0.7 g of NaOH beads (Sigma-Aldrich, #367176, St. Louis, MO, USA) that had been pre-washed with 350 μL acetonitrile/ACN and rinsed twice with 350 μL DMSO in a microfuge spin column (Thermo Fisher Scientific, #69705, Waltham, MA, USA). The permethylation reaction was run for 11 min with occasional gentle stirring. Samples were collected from the spin columns after centrifugation for 30 s at 1000× *g* and were promptly transferred into silanized glass tubes containing 0.5 M NaCl in sodium phosphate buffer (0.2 M, pH 7, 3.5 mL). The NaOH beads were washed twice with ACN (300 μL), and the liquid was added to silanized glass tubes. Samples were mixed with chloroform (1.2 mL) and then briefly centrifuged to remove of the upper aqueous layer, which was replaced with the aforementioned NaCl solution in sodium phosphate buffer. After three rounds of liquid/liquid (L/L) extraction, the ~1.2-mL chloroform layer was dried under nitrogen. Acid hydrolysis was performed by adding trifluoroacetic acid (TFA, Sigma-Aldrich, St. Louis, MO, USA, 2 M, 325 μL) to samples and incubating them at 121 °C for two hours. Samples dried under nitrogen were incubated for one hour at room temperature with freshly made sodium borohydride (10 mg/mL) in ammonium hydroxide (1 M, 475 μL) to reduce sugar aldehydes. Samples were mixed with methanol (MeOH, 63 μL) to remove excess borate and then dried under nitrogen, added to MeOH:acetic acid (9:1 *v*/*v*, 125 μL), and dried under nitrogen. Samples were fully dried in a vacuum desiccator for 20 min, and deionized water (18 μL) was added to dissolve any precipitates, followed by the addition of acetic anhydride (250 μL), sonication (2 min), and incubation (10 min, 60 °C). Samples were then incubated with concentrated TFA (230 μL) for 10 min at 60 °C. Samples were mixed with dichloromethane (1.8 mL) and deionized water (2 mL), followed by two rounds of L/L extraction (the ~1.8 mL organic layer was transferred to a silanized autosampler vial and dried under nitrogen) to clean up the samples for gas chromatography-MS. Samples were finally reconstituted in acetone (50 μL) and analyzed on an Agilent A7890 gas chromatograph equipped with a CTC PAL autosampler (Agilent Technologies, Santa Clara, CA, USA) coupled to a Waters GCT (time-of-flight: TOF) mass spectrometer (Milford, MA, USA).

Samples (1 μL) were injected at a 5:1 split ratio onto a 280 °C silanized glass liner (Agilent Technologies, #5183-4647, Santa Clara, CA, USA) fitted with a small plug of silanized glass wool. Chromatography was performed on a 30 m DB-5 ms GC column with helium as the carrier gas at a constant flow rate of 0.8 mL/min. The oven ramp was as follows: held at 165 °C for 0.5 min, then heated to 265 °C at 10 °C/min, followed by immediate heating to 325 °C at 30 °C/min with a final hold at 325 °C for three minutes. Electron ionization was carried out at 70 eV and 250 °C. Positive-ion mass spectra from individual TOF pulses over an m/z range 40–800 were summed every 0.1 s. The MS was calibrated daily with perfluorotributylamine to within an average mass accuracy of 10 ppm. Quanlynx 4.1 was used to sum extraction ion chromatograms (XICs) and integrate the resulting peaks, which were then manually checked for integration accuracy. A previously published list of XICs was employed to quantify each glycan node [26].

### 5.7. Hyaluronan Quantification and Enzymatic Stripping

The quantity of hyaluronan in EV samples was measured using a Hyaluronan Quantikine enzyme-linked immunosorbent assay (ELISA) Kit (R&D Systems, Inc., DHYALO, Minneapolis, MN, USA) according to the manufacturer’s instructions. All samples were analyzed in triplicate.

For the enzymatic digestion of surface hyaluronan in EV samples, a protocol adapted from [59] was used. Briefly, EVs (5 × 10^9^) were treated with 140 ng/mL hyaluronidase (Worthington Biochemical, 50592425, Lakewood, NJ, USA) in HES buffer (250 mM sucrose, 20 mM HEPES, and 1 mM EDTA, pH 5.32) for 4 h at 37 °C in a water bath, with gentle inversion every 15 min. Control samples were incubated with HES buffer without hyaluronidase. After the reaction, samples were placed on ice and processed through qEV (size exclusion chromatography columns) as previously described [60], and NTA was performed to determine the EV concentration.

### 5.8. Statistical Analysis

Data are presented as the mean ± standard deviation (SD) of at least triplicates (unless otherwise noted in the figure legends). Plotting and statistical analyses were performed using GraphPad Prism 9 software (GraphPad Software, San Diego, CA, USA). Statistical tests are indicated in the respective figure legends. Differences were considered statistically significant when *p* < 0.05.

## Data Availability

Raw data can be provided upon request.
